# Immunoglobulins G from Sera of Amyotrophic Lateral Sclerosis Patients Induce Oxidative Stress and Upregulation of Antioxidative System in BV-2 Microglial Cell Line

**DOI:** 10.3389/fimmu.2017.01619

**Published:** 2017-11-23

**Authors:** Milena Milošević, Katarina Milićević, Iva Božić, Irena Lavrnja, Ivana Stevanović, Dunja Bijelić, Marija Dubaić, Irena Živković, Zorica Stević, Rashid Giniatullin, Pavle Andjus

**Affiliations:** ^1^Center for Laser Microscopy, Department for Physiology and Biochemistry, Faculty of Biology, University of Belgrade, Belgrade, Serbia; ^2^Institute for Biological Research “Siniša Stanković”, University of Belgrade, Belgrade, Serbia; ^3^Institute for Medical Research, Military Medical Academy, Belgrade, Serbia; ^4^Immunology Research Centre “Branislav Janković”, Institute of Virology, Vaccines and Sera “Torlak”, Belgrade, Serbia; ^5^Clinic of Neurology, Clinical Center of Serbia, School of Medicine, University of Belgrade, Belgrade, Serbia; ^6^Department of Neurobiology, A.I. Virtanen Institute for Molecular Sciences, University of Eastern Finland, Kuopio, Finland; ^7^Laboratory in Neurobiology, Kazan Federal University, Kazan, Russia

**Keywords:** amyotrophic lateral sclerosis, immunoglobulin G, HyPer, SypHer, oxidative stress, antioxidative system, BV-2 microglia

## Abstract

Amyotrophic lateral sclerosis (ALS) is a neurodegenerative disorder with a very fast progression, no diagnostic tool for the presymptomatic phase, and still no effective treatment of the disease. Although ALS affects motor neurons, the overall pathophysiological condition points out to the non-cell autonomous mechanisms, where astrocytes and microglia play crucial roles in the disease progression. We have already shown that IgG from sera of ALS patients (ALS IgG) induce calcium transients and an increase in the mobility of acidic vesicles in cultured rat astrocytes. Having in mind the role of microglia in neurodegeneration, and a well-documented fact that oxidative stress is one of the many components contributing to the disease, we decided to examine the effect of ALS IgG on activation, oxidative stress and antioxidative system of BV-2 microglia, and to evaluate their acute effect on cytosolic peroxide, pH, and on reactive oxygen species (ROS) generation. All tested ALS IgGs (compared to control IgG) induced oxidative stress (rise in nitric oxide and the index of lipid peroxidation) followed by release of TNF-α and higher antioxidative defense (elevation of Mn- and CuZn-superoxide dismutase, catalase, and glutathione reductase with a decrease of glutathione peroxidase and glutathione) after 24 h treatment. Both ALS IgG and control IgG showed same localization on the membrane of BV-2 cells following 24 h treatment. Cytosolic peroxide and pH alteration were evaluated with fluorescent probes HyPer and SypHer, respectively, having in mind that HyPer also reacts to pH changes. Out of 11 tested IgGs from ALS patients, 4 induced slow exponential rise of HyPer signal, with maximal normalized fluorescence in the range 0.2–0.5, also inducing similar increase of SypHer intensity, but of a lower amplitude. None of the control IgGs induced changes with neither of the indicators. Acute ROS generation was detected in one out of three tested ALS samples with carboxy-H2DCFDA. The observed phenomena demonstrate the potential role of inflammatory humoral factors, IgGs, as potential triggers of the activation in microglia, known to occur in later stages of ALS. Therefore, revealing the ALS IgG signaling cascade in microglial cells could offer a valuable molecular biomarker and/or a potential therapeutic target.

## Introduction

Amyotrophic lateral sclerosis (ALS) is an adult-onset fatal neurodegenerative disease (1) characterized by loss of upper and lower motor neurons. Two clinically indistinguishable forms of ALS exist, sporadic (sALS) and familial (fALS), the latter comprising 5–10% of cases. Common hallmark of both sALS and fALS is neuroinflammation with astrogliosis, microgliosis, and infiltration of peripheral immune cells at the sites of neurodegeneration (2–6). Thus, non-neuronal cells play a crucial role in ALS, contributing to motor neuron death *via* non-cell autonomous mechanisms (7, 8).

Microglial cells originating from the myeloid lineage (9–11) are considered to be the resident mononuclear phagocytes in the central nervous system (9, 11, 12) that participate in the maintenance of tissue homeostasis and in immune defense of the brain (9, 12). In general, microglia contributes to the neuroinflammatory response by rapid morphological and functional changes which include phagocytosis, antigen presentation, production and secretion of reactive oxygen species (ROS), cytokines, and growth factors (13–16). In ALS, especially regarding the familial form of the disease, animal models have shown that microglial activation begins at or before disease onset (2) and the number of activated cells increases during the disease progression (17). However, the late phase of disease progression in an animal model was slowed by selective excision of human mutant superoxide dismutase 1 SOD1 or Cu/ZnSOD gene from microglia and macrophage lineages, even when neurons are expressing high levels of the mutant gene (17) and diminished expression of mutant gene in astrocytes delays microglial activation (18).

Although the majority of studies are done on animal models with overexpressed human genes characteristic for fALS, with the rationale that hallmarks of both fALS and sALS are similar. Nevertheless, there were some attempts to explicitly model the sporadic form of the disease. For this purpose, investigators have used either cerebrospinal fluid (CSF) or purified immunoglobulins G (IgG) from sporadic ALS patients, and evaluated either the viability or electrophysiological properties of neuronal cells treated with human CSF/IgG [for review see Ref. (19) and references therein]. The data on glial cells in such models of sALS emerged in the recent years, but the focus was mainly on astrocytes. ALS IgG were found to increase the mobility of endosomes and lysosomes of primary astrocytes, suggesting the involvement of endocytotic/autophagic pathways (20). In addition, intracellular calcium homeostasis of rat astrocytes was acutely affected by ALS IgG (21). On the other hand, treatment with sALS CSF caused disbalance in astrocytic cytokines, elevating production and release of proinflammatory, and decreasing anti-inflammatory cytokines and beneficial trophic factors, with impaired regulation of ROS, nitric oxide (NO), and glutamate (22). Elevated ROS and cellular peroxide levels, as well as increased mitochondrial SOD (MnSOD) activity were found in spinal cord extracts of pups intrathecally injected with sALS CSF (23), stressing out the role of oxidative stress. However, mice intraperitoneally injected with sALS IgG showed increased levels of both pro- and anti-inflammatory cytokines in the spinal cord, causing initial morphological and electrophysiological manifestations of degeneration, but with no actual death of spinal motor neurons (24). On the other hand in the study by Pullen et al. (25), ALS IgG inoculation caused motor neuron death, however with the different inoculation protocol and experimental time frame. These findings raise the question of the role of blood brain barrier impairment in ALS, with different manifestations of the leakage found in animal models and humans [for reviews see Ref. (19, 26)] that would favor entrance of humoral immune factors, e.g., IgG into the brain parenchyma, causing versatile effects on both neural and glial cells.

Oxidative stress has been assigned as one of the main causes of ALS (27–31), however, only a few studies on the ALS rat model could actually demonstrate a change in redox state *in vivo* (32). It was thus of particular interest to search for the missing link between the inflammatory humoral factors, ALS IgGs and oxidative stress. Having in mind the crucial role of microglia in onset and progression of the disease in animal models of ALS, we were interested in evaluating the extent of ALS IgG effect on this cell type. For that purpose, we acutely treated the BV-2 microglial cell line with ALS IgGs and measured their ROS response with fluorescence dyes, and monitored cytosolic peroxide and pH with genetically encoded sensors. In addition, the release of inflammatory factors and the expression of the antioxidative defense molecules has been checked by biochemical and molecular genetics assays. It has been shown that ALS IgGs do induce an acute effect on intracellular ROS production followed by cellular alkalization as well as a later induction of molecules of the inflammatory as well as of the antioxidant pathways.

## Materials and Methods

### Blood Samples and IgG Isolation

Blood samples were collected from clinically diagnosed sporadic (sALS) and familial (fALS) ALS patients and age-matched disease and healthy controls at the Institute of Neurology, Clinical Center of Serbia. Human sera from 20 ALS patients (3 fALS and 17 sALS; 13 males and 7 females) of age 59.6 ± 2.5 years (mean ± SEM) with the average score of 38.7 ± 1.0 on the ALS functional rating scale according to the El Escorial revised criteria for the diagnostics of ALS (33) and 11 controls (four healthy and seven disease controls; details in Table 1), age 57.4 ± 3.0 years, were collected for routine clinical examination with informed patient’s consent in accordance with The Code of Ethics of the World Medical Association (Declaration of Helsinki) for experiments involving humans. The protocol was approved by the Ethics committee of the Clinical Center of Serbia (No. 1985/5). A part of biological material was used for IgG isolation at the Institute of Virology, Vaccines and Sera-Torlak, Belgrade, Serbia as described previously (20).

**Table 1:** List of samples used in experiments.

Patient	Disease	ALSFRSr	Age	Sex	Used
#1	sALS	46	64	F	H
#2	sALS	45	64	M	H, B
#3	fALS (SOD 1 D90A)	45	69	M	X
#4	sALS	43	54	M	C, X
#5	sALS	42	42	M	I, E, B
#6	sALS	42	71	F	X
#7	sALS	41	67	M	H
#8	sALS	41	65	M	H
#9	sALS	40	62	M	H, B
#10	sALS	40	60	M	C, I, X
#11	sALS	38	70	M	H
#12	sALS	37	50	M	E, B
#13	sALS	37	67	F	X
#14	fALS (C9orf72)	36	34	F	H, B
#15	sALS	36	41	M	H
#16	sALS	35	71	M	C, I, X
#17	sALS	34	68	F	E, B
#18	sALS	34	62	F	H, B
#19	sALS	33	46	F	H, B
#20	fALS (C9orf72)	28	64	M	H, B
#21	Healthy	–	57	F	B
#22	Healthy	–	57	F	H
#23	Healthy	–	49	M	H, B
#24	Healthy	–	63	M	H, B
#25	Polyneuropathy	–	67	M	E, B
#26	Polyneuropathy	–	41	M	E, B
#27	Polyneuropathy	–	74	M	X
#28	Polyneuropathy	–	64	M	C, I, X
#29	Nonspecific ataxia	–	58	M	I, X
#30	Discopathy of cervical spine	–	44	M	H
#31	Discopathy of thoracal spine	–	57	F	H

*Patients are scored on the ALS revised functional rating scale (ALSFRSr) reflecting the stage of the disease (lower scores indicate disease progression). sALS, sporadic ALS; fALS, familial ALS (with type of mutation indicated in the brackets). Labels of experiments in which particular samples were used are: H, HyPer&SypHer; C, carboxy-H2DCFDA; I, immunocytochemistry; E, ELISA; X, expression (RT-qPCR); B, biochemistry*.

### Cell Culture and Treatments

In this study, we used microglial BV-2 cell line, frequently used as substitute for primary microglia, derived from raf/myc-immortalized murine neonatal microglia (34). Cells were used in passages 5–15. BV-2 cell line was maintained in growth medium consisting of RPMI with l-glutamine, and sodium pyruvate (Sigma Aldrich, Germany), supplemented with 25 mM HEPES, 10% fetal bovine serum (Gibco, Invitrogen, USA), and penicillin/streptomycin (Gibco, Invitrogen, USA) at 37°C in humidified incubator with 5% CO_2_. After the cells reached ~90% confluence, they were washed with PBS, trypsinized (0.25% trypsin and 0.02% EDTA), centrifuged (500 *g*, 5 min), and then passaged and/or plated for the experiments. For biochemical analysis, cells were plated on 24-well plates (6 × 10^4^ cells/well) in 0.5 ml of growth media, while for the expression analysis, BV-2 microglial cells were plated on 6-well plates (3 × 10^5^ cells/well) in 2 ml of growth media. On the following day, the culture medium was replaced with the culture medium containing ALS IgG (0.1 mg/ml) or control IgG (0.1 mg/ml) and cells were kept for the indicated period of time (4 or 24 h). The IgG concentration tested was selected from our previous studies on neuronal and glial cells in culture (20, 21, 35). Cells kept in medium without IgG were used as a control.

### Measurement of NO Production

BV-2 cells (6 × 10^4^ cells/well) were plated and treated for 24 h (here and in Sections “Malondialdehyde (MDA) Determination” to “Total Glutathione Determination,” at least in duplicate for each IgG) as described. After the indicated time supernatants were discarded, cells were washed in ice-cold PBS then collected with plastic scraper, lysed by sonication and centrifuged at 15,000 *g* for 5 min at 4°C for NO determination. NO is a highly unstable molecule and therefore the production was determined indirectly by measuring nitrite concentration spectrophotometrically at 492 nm using Griess method, after reducing nitrates to nitrites by cadmium reduction (36). Results are expressed as mean nitrite concentration (μM) ± SEM for each group.

### Malondialdehyde (MDA) Determination

BV-2 cells (6 × 10^4^ cells/well) were plated and treated for 24 h and collected for measurements as described. MDA content was determined by the spectrophotometric method of Vilicarra et al. (37). First, thiobarbituric acid (TBA) reagent (15% trichloracetic acid and 0.375% TBA water solution, Merck-Darmstadt, Germany) was mixed with cell lysate, heated to 95°C, centrifuged, and then the absorbance was measured at 532 nm. Results are expressed as mean MDA concentration (nmol/ml) ± SEM for each group.

### SOD Activity Determination

BV-2 cells (6 × 10^4^ cells/well) were plated and treated for 24 h, then collected for measurements as described. Total SOD activity, which combines the activity of two isoforms, MnSOD and cytoplasmic SOD (Cu/ZnSOD), was determined by epinephrine method. SOD activity was determined by spectrophotometric measurement of a decrease in the rate of the spontaneous epinephrine autooxidation at 480 nm. The kinetics of enzyme activity was followed in 50 mM carbonate buffer (pH 10.2) with 1 mM EDTA after the addition of 10 mM epinephrine and 5 mM KCN for MnSOD (38). Cu/ZnSOD activity was determined as a difference between total SOD and MnSOD activity. Results are presented as units per milligram of total protein (U/mg). One unit is described as an amount of protein (enzyme) required for 50% inhibition of autooxidation of epinephrine. Total protein concentration was determined by Lowry method (39).

### Catalase (CAT) Activity Determination

Catalase activity was assayed by a method based on spectrophotometric determination of colored complex formed between ammonium molibdate and H_2_O_2_ at 405 nm (40). The results are expressed as units per milligram of total protein (U/mg), whereas the unit represents an amount of H_2_O_2_ reduced per min (μM H_2_O_2_/min).

### Glutathione Peroxidase (GPx) Activity Determination

Glutathione peroxidase activity was assayed by indirect method that is based on spectrophotometric measurement of NADPH oxidation by glutathione reductase (GR) at 340 nm (41, 42). Briefly, GPx catalyzes the oxidation of GSH to GSSG which is then recycled back to GSH by GR. In that process GR reduces NADPH coenzyme, as a donor of reducing equivalents, to NADP^+^. The results are expressed as units per milligram of proteins (U/mg), whereas one unit represents an amount of reduced NADPH per min (μM NADPH/min).

### GR Activity Determination

Activity of GR was determined using method that is based on fluorimetric measurement of GR-mediated NAPDH oxidation to NADP^+^ (43). Decrease in NADPH fluorescence was measured with excitation/emission of 360/460 nm. We used 100 mM NAD^+^ as a standard in the reaction. Results are expressed as units per milligram of proteins (U/mg). One unit is described as an amount of oxidized NAPDH per min (μM NADPH/min).

### Total Glutathione Determination

BV-2 cells (6 × 10^4^ cells/well) were plated and treated for 24 h, then collected for measurements as described. Intracellular glutathione content was assessed by DTNB-GSSG reductase recycling assay (44). The formation of 5-thio-2-nitrobenzoic acid (TNB), which is proportional to total glutathione content in the sample, was measured spectrophotometrically at 412 nm for 6 min. Total glutathione concentration in samples was determined from standard curve constructed with known GSSG concentrations. Results are expressed as mean total glutathione concentration (nmol/ml) ± SEM for each group.

### Quantitative Real-time PCR

BV-2 cells (3 × 10^5^ cells/well) were plated, treated for 4 h with ALS IgG or control IgG (in duplicate for each IgG) and then collected. Total mRNA isolation was performed by using TRIzol reagent (Invitrogen, USA). The RNA content was quantified spectrophotometrically at 260 nm and 1 µg of RNA was used for reverse transcription with High Capacity cDNA Reverse Transcription Kit (Applied Biosystems, Foster City, CA, USA). Real-time PCR amplifications were performed with SensiFAST™ SYBR^®^ Hi-ROX Kit (Bioline, United Kingdom) and specific primers (sequences and annealing temperatures are given in Table 2, Invitrogen, USA), with QuantStudio TM Real-Time PCR System (Applied Systems, USA). The results were analyzed by using 2^−ΔΔCt^ method. In previous experiments on BV-2 cells it was shown that there is no change in GAPDH, ACTB, and HPRT gene expression following LPS stimulation of these cells (Bozic, unpublished), therefore we used GAPDH and ACTB genes expression as internal controls. Since there were no differences, only results with GAPDH gene expression as an internal control are presented.

**Table 2:** List of primers used for real-time PCR.

Target gene	Reverse primer	Forward primer	Annealing T (°C)
MnSOD	CAGACCTGCCTTACGACTATGG	CTCGGTGGCGTTGAGATTGTT	60
CAT	AGCGACCAGATGAAGCAGTG	TCCGCTCTCTGTCAAAGTGTG	60
GPx	AGTCCACCGTGTATGCCTTCT	GAGACGCGACATTCTCAATGA	60
NOX2	GGGAACTGGGCTGTGAAT	CAGTGCTGACCCAAGGAGTT	60
NHE1	GCCTCATGAAGATAGGTTTCCA	ACGTCTGATTGCAGGAAGGG	60
TNF-α	CTGAACTTCGGGGTGATCGG	GGCTTGTCACTCGAATTTTGAGA	60
GAPDH	GTTGTCTCCTGCGACTTCA	TGGTCCAGGGTTTCTTACTC	60

### Enzyme-Linked Immunosorbent Assay (ELISA)

BV-2 cells (6 × 10^4^ cells/well) were seeded and treated with ALS IgG or control IgG (in triplicate for each IgG) for 24 h as previously described. After the indicated time supernatants were collected and TNF-α concentration was determined by ELISA by using detection and capture antibody (eBioscience, Germany) according to the manufacturer’s protocol. After applying biotinylated capture antibody, avidine-HRP complex was added, and subsequently chromogenic substrate 3,3′,5,5′-tetramethylbenzedine (eBioscience, Germany). The reaction was stopped by adding 1 M H_3_PO_4_ and absorbance was measured at 450 nm. TNF-α concentration was determined from standard curve constructed with known murine recombinant TNF-α concentrations. Results are expressed as mean concentration (pg/ml) ± SEM for each group.

### Immunocytochemistry and Confocal Microscopy

BV-2 cells were plated onto PLL-coated 10 mm circular glass coverslips (1.5 × 10^4^ per coverslip). On the following day, cells were treated with human IgG (0.1 mg/ml, ALS or control, in growth media; in duplicate for each IgG) for additional 24 h. After treatment, cells were briefly washed with PBS, fixed in 4% PFA for 15 min, and washed in PBS. Then, the cell membrane was labeled with Wheat Germ Agglutinin, Tetramethylrhodamine Conjugate (WGA, 1:2,000; Molecular Probes, Thermo Fisher Scientific) for 10 min. Next, cells were permeabilized with 0.05% Triton X-100 for 15 min, following washing in PBS. Cells were then labeled with secondary antibody, goat anti-human IgG AlexaFluor 633 (1:200, Molecular Probes, Thermo Fisher Scientific) for 1 h in the dark at 37°C. After several washing steps with PBS, coverslips were incubated for 10 min with a nuclear counterstain (DAPI, 1:4,000), washed again thoroughly, and mounted on microscopic slides using Mowiol 4-88 mounting medium (Sigma Aldrich, Germany). Control staining was done by omitting IgG from the treatment.

Stained cells were visualized on a confocal laser-scanning microscope (LSM 510, Carl Zeiss GmbH, Jena, Germany) equipped with HeNe (543 and 633 nm) lasers. Oil-immersion objective 63×/NA 1.4 was used, and pinhole was set to 2.2 µm for both detection channels.

### ROS Imaging and Data Analysis

For live cell imaging BV-2 cells were seeded onto 7 mm (6 × 10^3^ cells) or 10 mm (1.5 × 10^4^ cells) round glass coverslips (Menzel Glasser, Germany), coated with poly-l-lysine (50 µg/ml, Sigma Aldrich, Germany). For each IgG sample 1–4 coverslips were prepared. The number of analyzed cells [region of interest (ROI)] is indicated in figures. In order to evaluate acute hydrogen peroxide production in response to IgG treatment, BV-2 cells were transfected with HyPer, a genetically encoded sensor for H_2_O_2_ with expanded dynamic range (45), pC1-HyPer-2 (Addgene plasmid # 42211). Since HyPer shows pH dependence, a set of control experiments were done with BV-2 cells transfected with SypHer, peroxide-insensitive version of HyPer (46), pC1-HyPer-C199S (Addgene plasmid # 42213). Transfection was done with Lipofectamine (LTX, Invitrogen, USA), or Turbofect (Thermo Fisher Scientific, USA), 24 h after plating, and cells were imaged on the following day. In addition, 6-carboxy-2′,7′-dichlorodihydrofluorescein diacetate (carboxy-H2DCFDA, Molecular Probes, Thermo Fisher Scientific, USA) a general oxidative stress indicator was used to detect acute generation of ROS following treatment with IgG. BV-2 cells (24–48 h after plating) were loaded with 50 µM carboxy-H2DCFDA in working solution for 30 min. Cells were briefly rinsed three times and imaged immediately.

Transfected or dye-loaded coverslips were transferred into the recording chamber supplied with 1 ml of working solution, placed on an inverted epifluorescent microscope (AxioObserver A1, Carl Zeiss, Germany) equipped with water, glycerin, and oil immersion objective LD LCI Plan-Apochromat 25x/0.8 (Carl Zeiss, Germany) and combined with VisiFluor Imaging System. The excitation light source was Xenon Short Arc lamp (Ushio, Japan) combined with high speed polychromator system (VisiChrome, Visitron Systems, Germany). The excitation light (480 nm) and the emission light passed through the FITC filter set (Chroma Technology Inc., USA). Time-lapse images were obtained by “evolve”-EM 512 Digital Camera System (Photometrics, USA) *via* VisiView high performance imaging software (Visitron Systems, Germany). Initially, fluorescence intensities were recorded for 2–5 min to determine the baseline fluorescence (*F*_0_). Thereafter, human IgG (0.1 mg/ml) in working solution (1 ml) were applied directly to the imaged cells by customized delivery system, *via* glass pipettes (0.8 mm inner diameter, positioned ~350 μm away and ~1 mm above the cells, at the angle of 45°) connected to High Speed Solution Exchange System (ALA Scientific Instruments, USA) with pinch valves and VC3 electronic valve controller. The volume in the recording chamber was kept at ~1 ml by suction from the top of solution. The response of cells to IgG was recorded for additional 5–10 min, followed by several minutes of constant perfusion of working solution (washing step), after which the cells were stimulated by H_2_O_2_ (100 µM or 4 mM) for 3–10 min, and washed again with working solution. In another set of experiments after recording fluorescence intensities for 2–5 min, 30 mM NH_4_Cl in working solution was applied for 4 min and then washed with working solution for several minutes.

The working solution consisted of NaCl (152 mM), KCl (2.5 mM), CaCl_2_ (2 mM), MgCl_2_ (1 mM), d-glucose (10 mM), and HEPES (10 mM), pH 7.4, adjusted with NaOH. The osmolality of solution was ~300 mOsM, measured by vapor pressure osmometer (Vapro 5520, Wescor, USA).

After the experiment, the analysis began with extracting average fluorescence intensities from ROI that were drawn around each cell, in each time point of the experiment. Additionally, several ROIs were extracted from the background. The background correction was done by subtracting averaged background fluorescence from each ROI in every time point. Time sets of data from each ROI, corrected for background were further normalized to the baseline fluorescence (*F*_0_). Normalized fluorescence data, were expressed as Δ*F*/*F*_0_, where Δ*F* represents the change in fluorescence emission from the baseline fluorescence.

### Statistical Analyses

Effects of ALS IgG and control IgG on NO, MDA and GSH levels, antioxidative enzymes gene expression and activities, NADPH oxidase 2 (NOX2) and Na^+^/H^+^ exchanger 1 (NHE1) gene expression and TNF-α gene expression and release were analyzed by comparing the means (absolute values) by one-way ANOVA, followed by Bonferroni *post hoc* test, with a significance level of *p* < 0.05. Acute effects of ALS and control IgG on ROS, H_2_O_2_ and pH were analyzed by comparing the amplitudes in certain time points (normalized fluorescence − Δ*F*/*F*_0_) by two-tailed Student’s *t*-test, with a significance level of *p* < 0.05. All mean values were presented as ±SEM.

## Results

### Production of Markers of Oxidative Stress (NO and MDA) Is Increased by ALS IgG in BV-2 Cells

**Figure 1:**
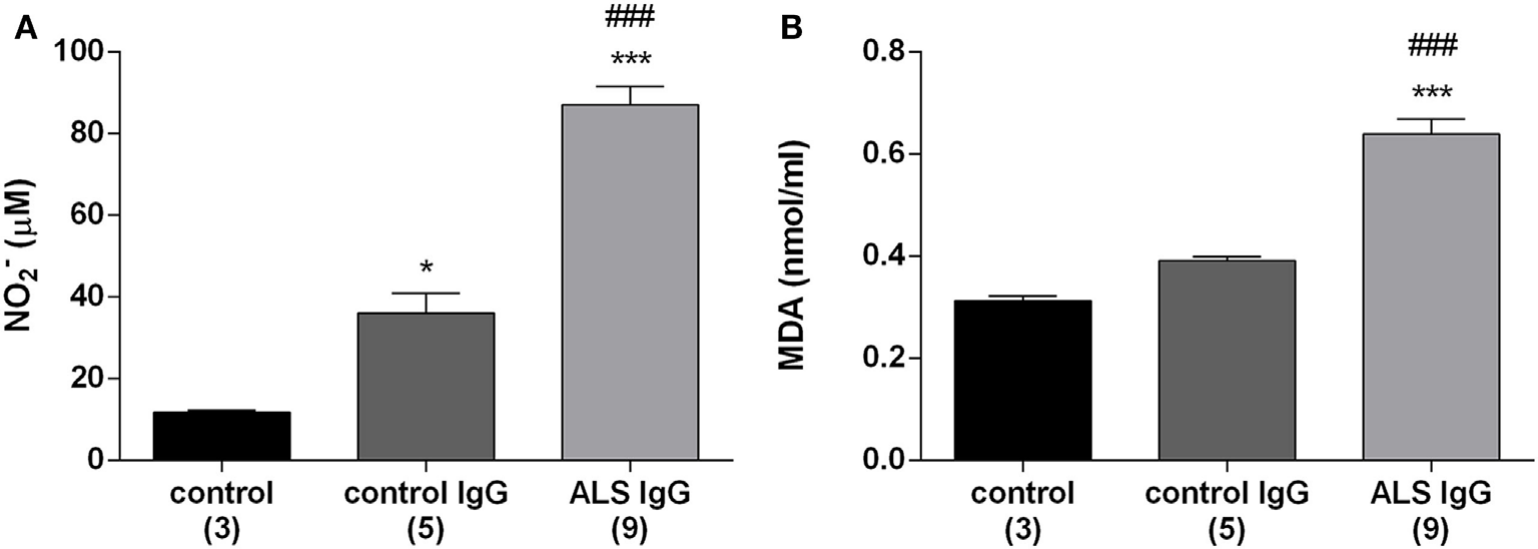
Immunoglobulin G (IgG) isolated from amyotrophic lateral sclerosis (ALS) patients increase the production of nitric oxide (NO) and malondialdehyde (MDA) in BV-2 cells. NO production **(A)** and MDA production in BV-2 cells **(B)** were measured 24 h after treatments with control IgG and ALS IgG (0.1 mg/ml). NO production was measured indirectly using Griess assay and is expressed as NO_2_^−^ concentration. The results for each group are presented as mean ± SEM; ***p < 0.001 compared with untreated control group, ^###^*p* < 0.001 compared with the group treated with control IgG. The numbers in brackets indicate the number of different control IgG and ALS IgG samples examined and the number of untreated controls.

Production of NO and the intracellular level of MDA were analyzed as they are relevant oxidative stress indicators. MDA is an end product of cell membrane phospholipids degradation and therefore is used as an index of lipid peroxidation (47). In this set of experiments, we treated BV-2 cells with ALS IgG isolated from nine different patients (seven sALS and two fALS) with an average ALSFRSr 36.6 ± 1.7, aged 54.7 ± 4.0 years and with IgG from age-matched control subjects (three healthy and two disease controls, Table 1), and 24 h after the treatments cell lysates were collected for further examination. As shown in Figure 1A both ALS IgG and control IgG treatments resulted in a significant increase of NO production relative to untreated control (87.13 ± 4.52 and 36.07 ± 4.86 relative to 11.69 ± 0.53 µM; *p* < 0.001 and *p* < 0.05, respectively). Furthermore, ALS IgG caused 2.4-fold higher NO production than control IgG (*p* < 0.001). Similarly, treatment with ALS IgG induced a higher level of MDA compared with untreated cells and cells treated with control IgG (0.64 ± 0.03 relative to 0.31 ± 0.01 and 0.39 ± 0.01 nmol/ml, both *p* < 0.001; Figure 1B).

### ALS IgG Enhance the Antioxidative System in BV-2 Cells

**Figure 2:**
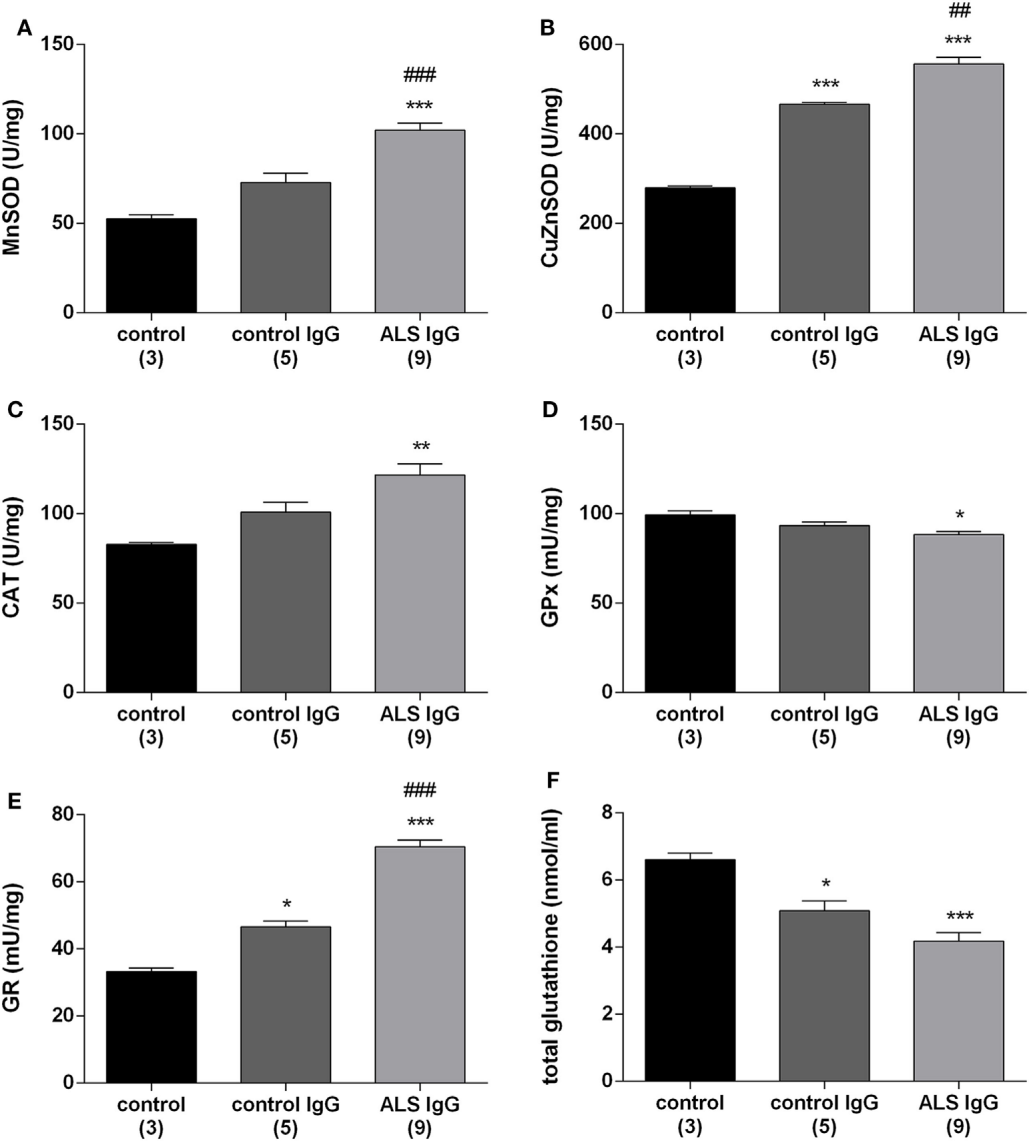
Immunoglobulin G (IgG) isolated from amyotrophic lateral sclerosis (ALS) patients increase antioxidative enzymes activities and decrease total glutathione content in BV-2 cells. Mitochondrial superoxide dismutase (MnSOD) **(A)**, cytosolic SOD (Cu/ZnSOD) **(B)**, catalase (CAT) **(C)**, glutathione peroxidase (GPx) **(D)**, glutathione reductase (GR) activity **(E)**, and total glutathione content in BV-2 cells **(F)** were measured 24 h after ALS IgG and control IgG treatments (0.1 mg/ml). The results of antioxidative enzymes activities for each group are presented as mean specific activities per milligram of total proteins (U/mg) ± SEM and total glutathione content is presented as mean concentration in nmol/ml ± SEM; **p < 0.01 ***p < 0.001 compared with untreated control group, ^#^*p* < 0.05, ^###^*p* < 0.001, compared with the group treated with control IgG. The numbers in brackets indicate the number of different control IgG and ALS IgG samples examined and the number of untreated controls.

To examine the effect of IgG on the antioxidative system in BV-2 cells, we measured the activity of antioxidative enzymes (MnSOD, Cu/ZnSOD, CAT, GR, GPx) as well as the total intracellular glutathione content. BV-2 cells were treated for 24 h with ALS IgG from nine different patients and age matched controls (all same as above, Table 1). Results show that MnSOD and Cu/ZnSOD activities were significantly elevated, in ALS IgG-treated cells compared with untreated control (102.18 ± 3.98 vs. 52.53 ± 2.27 U/mg and 556 ± 15.12 vs. 279.64 ± 3.92 U/mg, respectively, both *p* < 0.001). In addition, both enzymes’ activities were significantly higher in ALS IgG-treated compared with control IgG-treated cells (1.4-fold, *p* < 0.001 and 1.2-fold, *p* < 0.01, respectively; Figures 2A,B). Similarly, CAT activity was increased in BV-2 cells treated with ALS IgG (121.72 ± 6.14 U/mg, that was 1.5-fold higher as compared with the control group, 82.78 ± 1.18 U/mg, *p* < 0.01, and 1.2-fold higher as compared to cells treated with control IgG, 100.85 ± 5.41 U/mg, but with no statistical significance; Figure 2C). On the other hand, we observed a decreased GPx activity after ALS IgG treatment in comparison with the untreated group (88.42 ± 1.79 vs. 99.28 ± 2.46 U/mg, *p* < 0.05), however, we did not observe a significant difference from the activity in control IgG (93.35 ± 2.11 U/mg), all shown in Figure 2D. IgG treatments (ALS and control) also increased GR activity compared with the control group (33.33 ± 1.03 U/mg, *p* < 0.001 and *p* < 0.05, respectively), while ALS IgG induced a 1.5-fold higher activity (70.44 ± 2.04 U/mg) than control IgG (46.58 ± 1.8 U/mg, *p* < 0.0001), all shown in Figure 2E. Finally, we measured total intracellular glutathione content and found that it was decreased after both ALS IgG (4.18 ± 0.25 nmol/ml) and control IgG treatments (5.09 ± 0.29 nmol/ml) compared with the untreated group (6.61 ± 0.21 nmol/ml, *p* < 0.001, *p* < 0.05, respectively). Although there was no statistical significance, there was a trend of a decrease in total glutathione content after ALS IgG treatment compared with control IgG treatment (Figure 2F).

### Expression of Genes for Antioxidative Enzymes, NOX2 and NHE1 following ALS IgG Treatment

**Figure 3:**
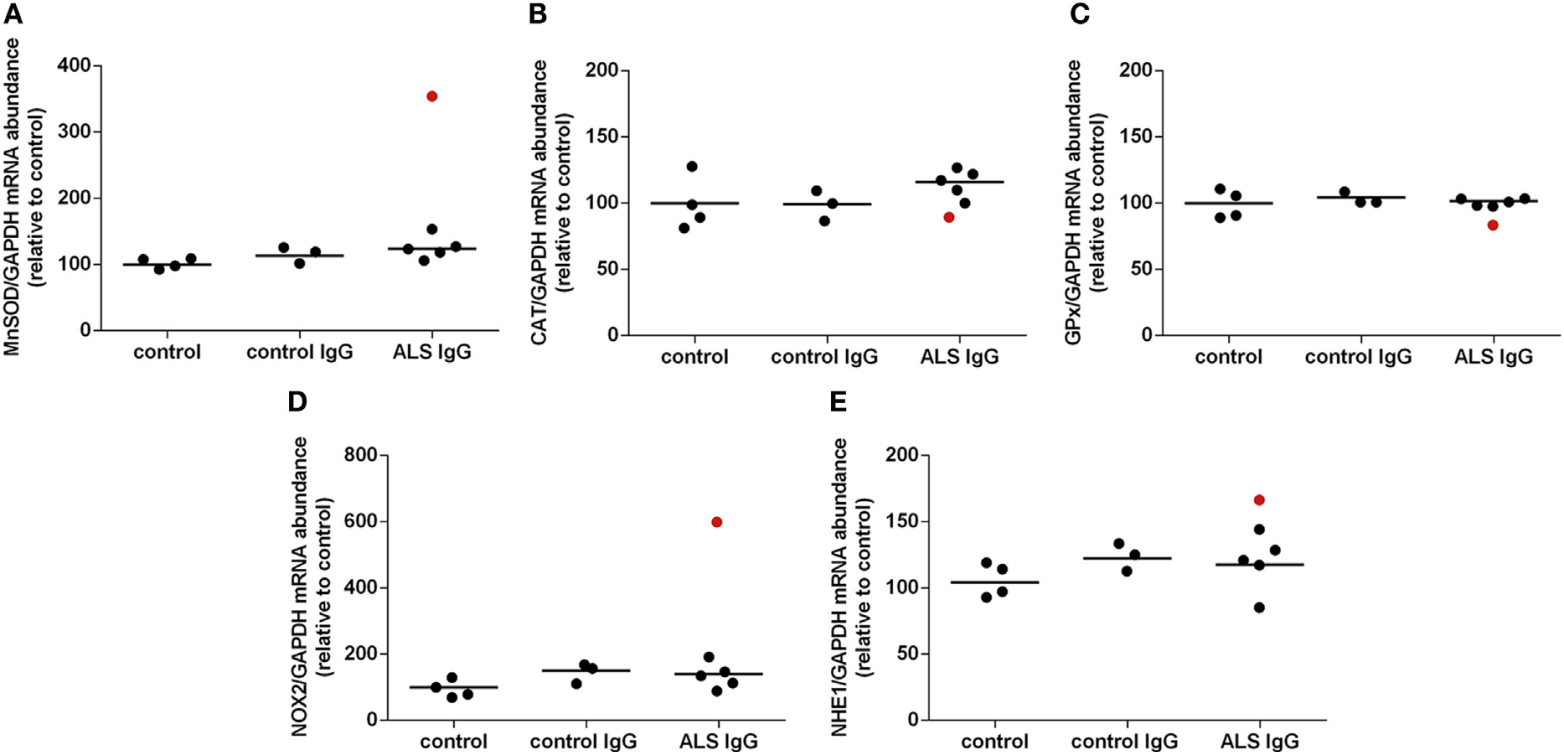
Effect of amyotrophic lateral sclerosis (ALS) immunoglobulin G (IgG) on gene expression of antioxidative enzymes, NOX2, and NHE1 in BV-2 cells. Expression of mitochondrial superoxide dismutase (MnSOD) **(A)**, catalase (CAT) **(B)**, glutathione peroxidase (GPx) **(C)**, NOX2 **(D)**, and NHE1 **(E)** was determined by RT-qPCR following 4 h ALS IgG or control IgG treatment. Untreated cells were used as a control. mRNA expression of these genes was expressed relative to the GAPDH gene expression, as an internal control. The data represent gene expression obtained for each IgG and untreated control. Red dot in ALS IgG group represents results obtained after treatment with ALS IgG from a fALS patient (sample #3; see Table 1) and is not included in calculation of mean value (line). Other ALS samples used were #4, #6, #10, #13, and #16 (see Table 1).

To further examine the effect of ALS IgG on the antioxidative system of BV-2 cells, we determined the gene expression levels for MnSOD, CAT, and GPx by qRT-PCR. BV-2 cells were treated with human IgG and the mRNA content was determined 4 h after the treatment (Figure 3). In this set of experiments, we used IgG from 6 different ALS patients (ALSFRSr 40.3 ± 1.5, aged 65.3 ± 2.8 years) and IgG from three disease controls (aged 65.3 ± 4.7 years, Table 1). Analysis of the results shown in Figure 3A indicates that neither ALS IgG nor control IgG induced significant changes in MnSOD expression compared with untreated control. Interestingly, MnSOD gene expression was only upregulated (approx. 3.5-fold) after treatment with ALS IgG from a patient having fALS with ALSFRSr 45 (sample #3 in Table 1). Following a 4 h ALS IgG treatment, there were also no significant changes in expression of CAT and GPx genes (Figures 3B,C).

Furthermore, we wanted to examine the effect of ALS IgG on the gene expression for NOX2, an enzyme that produces O_2_^−^ and therefore is a potential oxidative stress inducer. We did not observe any notable difference in gene expression after ALS IgG treatment when compared to either control IgG treated or untreated control group. However, ALS IgG from the fALS patient with ALSFRSr 45 (sample #3 in Table 1) induced an approx. threefold increase in NOX2 gene expression as compared to control IgG (Figure 3D), resembling the effect on MnSOD gene expression (Figure 3A). Finally, we analyzed the effect of ALS IgG on gene expression of NHE1which may be a regulator of NOX2 activity (48), however, we did not find any significant alterations when compared either with control IgG or the untreated control.

### ALS IgG Modulates the Release of TNF-α

**Figure 4:**
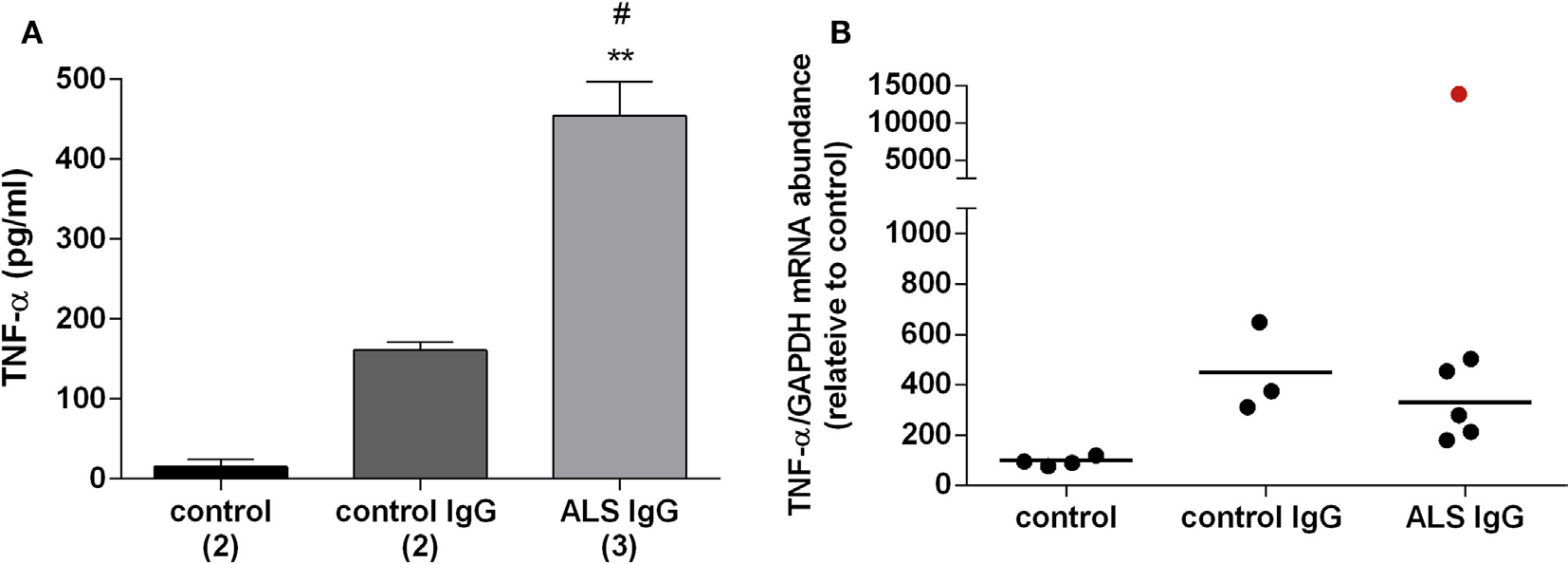
Effect of amyotrophic lateral sclerosis (ALS) immunoglobulin G (IgG) on release and gene expression of proinflamatory cytokine TNF-α. **(A)** BV-2 cells were treated with ALS IgG or control IgG for 24 h. Levels of TNF-α in culture medium after applied treatments were determined by enzyme-linked immunosorbent assay (ELISA). Culture medium from untreated cells was used as a control. Data are presented as mean TNF-α (pg/ml) ± SEM for each group. The numbers in brackets indicate the number of different control IgG and ALS IgG samples examined and the number of untreated controls. Significance level showed inside the graphs: **p < 0.01 compared with untreated control group, ^#^*p* < 0.05 compared with the group treated with control IgG **(B)** mRNA expression of TNF-α gene after 4 h ALS IgG or control IgG treatment was determined by RT-qPCR and expressed relative to the GAPDH gene expression. Untreated cells were used as a control. The data are represented as individual values obtained for each IgG and control. Red dot represents gene expression of TNF-α after treatment with ALS IgG from fALS patient (sample #3; see Table 1) and is not included in mean value (line). Other ALS samples used were #4, #6, #10, #13, and #16 (see Table 1).

The inflammatory potential of ALS IgG was evaluated by determining their effect on the TNF-α release and gene expression following 24 and 4 h treatments, respectively, using the same samples as in evaluating activity of antioxidative enzymes. ALS IgG-treatment induced a rise in TNF-α release (454.31 ± 42.75 pg/ml) compared with control IgG-treated group (160.88 ± 10.37 pg/ml, *p* < 0.05) as well as with the untreated group (15.15 ± 9.76 pg/ml, *p* < 0.01), as seen in Figure 4A. The results of the TNF-α gene expression experiments showed no difference between the groups. Interestingly, ALS IgG from the fALS patient with ALSFRSr 45 (sample # 3 in Table 1) caused a marked increase in TNF-α gene expression (Figure 4B).

### Human IgG Colocalize with Membrane of BV-2 Cells after 24 h Treatment

**Figure 5:**
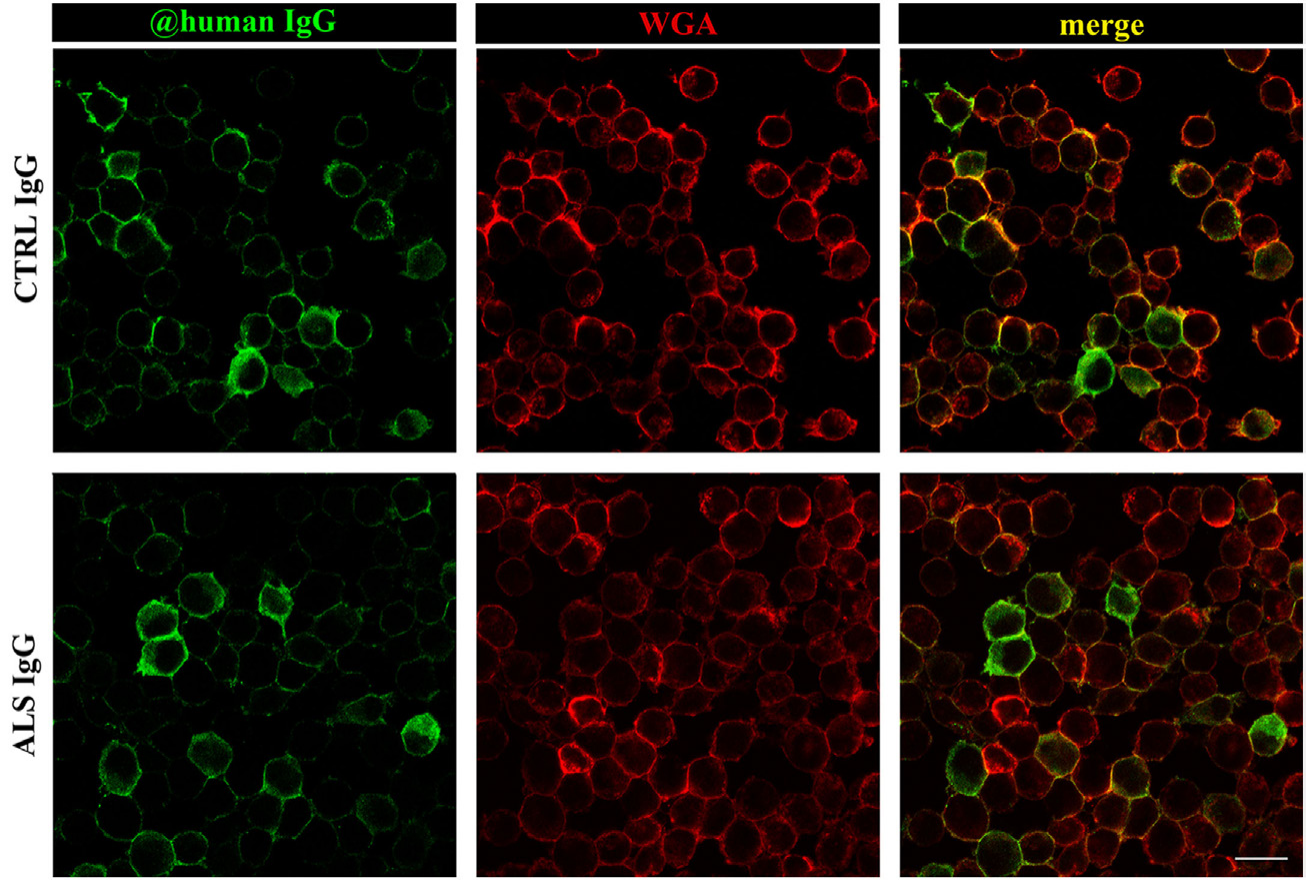
Immunocytochemical localization of immunoglobulin G (IgG) after 24 h treatment of BV-2 cells. BV-2 cells were treated with amyotrophic lateral sclerosis (ALS) (sample #16; second row) or control IgG (sample #28; first row) for 24 h. After the treatment, cells were fixed and labeled with WGA (red; membrane marker) and anti-human IgG (green). Colocalization of both markers is indicated by yellow in the merge column. Scale bar 20 µm.

Previous results have shown that ALS IgG increase the markers of oxidative stress, but also enhance the antioxidative system in BV-2 cells after 24 h treatment. Although control IgGs differ in effect from ALS IgG, the treatment with control IgG increased NO production, as well as the activity of Mn and Cu/ZnSOD and GR, and decreased total glutathione content. Since both ALS and control IgGs affected BV-2 cells after 24 h treatment, we were interested to evaluate the localization of human IgG in BV-2 cells following 24 h incubation. For this purpose, cells were fixed after treatment with human IgG (three ALS, two control IgG, details in the Table 1), and stained with WGA, the membrane marker, while IgGs were visualized with fluorescent secondary antibody against human IgG. Interestingly, both ALS and control IgGs showed similar staining pattern on BV-2 cells, and these humoral immune factors could be visualized mostly on the cellular membrane (Figure 5). We did not detect any substantial changes in the shape of BV-2 cells in any of the treatments compared to the untreated control (data not shown). The cells were mostly of round morphology in the middle focal plane (as represented in the micrographs of Figure 5).

### ALS IgG Acutely Increase the pH of BV-2 Cells Which Is Accompanied with Elevated Cytoplasmic Peroxide

**Figure 6:**
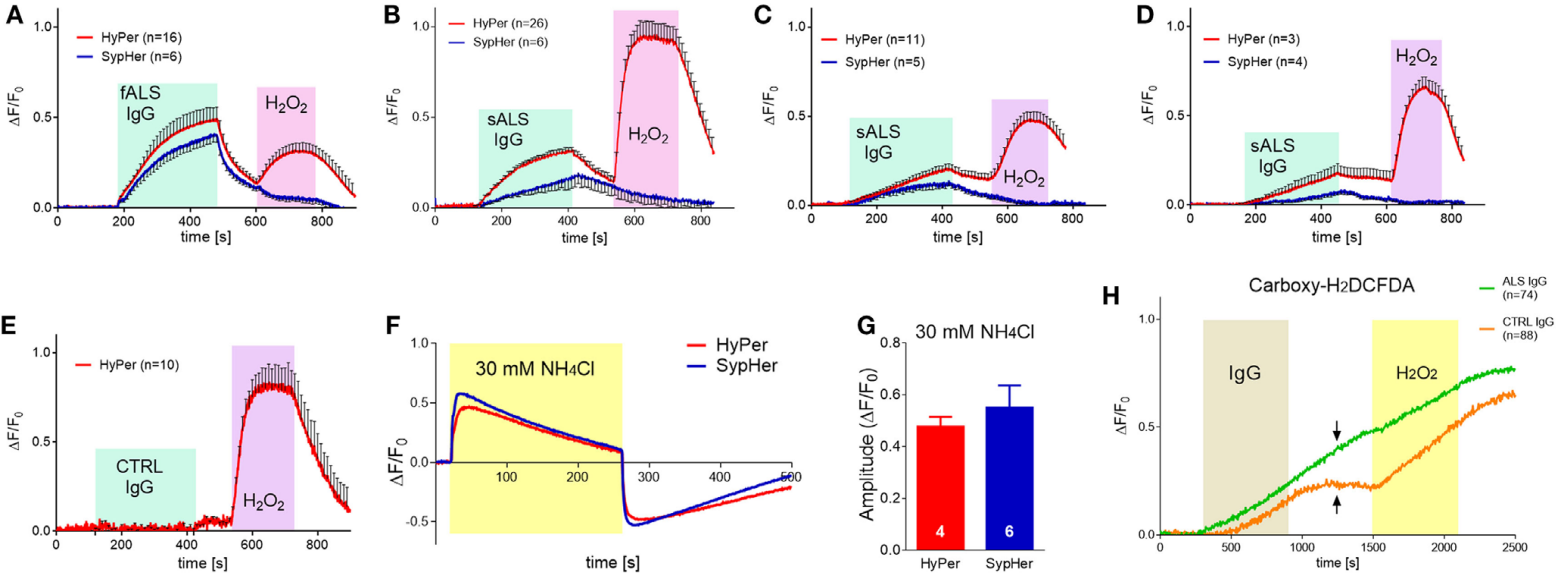
Accute effect of amyotrophic lateral sclerosis (ALS) immunoglobulin G (IgG) on pH change, peroxide production, and reactive oxygen species (ROS) generation in BV-2 cells. **(A–F)** BV-2 cells were transfected with HyPer (red trace; peroxide and pH sensitive) or SypHer (blue trace; pH sensitive). **(A–D)** Averaged fluorescence of BV-2 cells treated with ALS IgG for 5 min (indicated by cyan box) followed by washing and treatment with 100 µM H_2_O_2_ (indicated by magenta box). HyPer transfected cells show rise in fluorescence intensity when challenged both by ALS IgG and H_2_O_2_, while SypHer transfected cells increase the fluorescence intensity when challenged with ALS IgG only. The difference in ALS IgG treated HyPer and SypHer traces is significant in panel B. Number of cells is indicated in brackets. Samples #14 **(A)**, #7 **(B)**, #11 **(C)**, and #9 **(D)** were used for treatments. **(E)** Averaged fluorescence of HyPer transfected BV-2 cells treated with control IgG (sample #30) for 5 min (indicated by cyan box), followed by washing and treatment with 100 µM H_2_O_2_ (indicated by magenta box). **(F)** Representative traces of HyPer and SypHer transfected BV-2 cells treated with 30 mM NH_4_Cl (indicated by yellow box). **(G)** Comparison of the amplitudes of maximal fluorescence increase of HyPer and SypHer transfected BV-2 cells treated with 30 mM NH_4_Cl. Both plasmids show similar sensitivity to pH change. Number of cells is indicated in the base of the histogram bars. **(H)** BV-2 cells were loaded with Carboxy-H2DCFDA, general ROS indicator. Graph shows averaged traces of cell responses to ALS IgG (sample #16; green trace) or control IgG (sample #28; orange trace) for 10 min, followed by washing step and treatment with 4 mM H_2_O_2_. The time of IgG treatment (0.1 mg/ml) is indicated by the gray box, while yellow box designates H_2_O_2_ action. Number of cells is indicated in the brackets. Note that fluorescence continues to increase even during the wash period in the case of ALS IgG, in contrast to control IgG, where it remains on the same level (indicated by arrows).

All the effects of ALS IgGs so far were evaluated after 4 or 24 h treatments. Hence, we were interested to see if there are any acute effects of human IgG on BV-2 cells. For this purpose, we used an epifluorescent imaging system, and plasmids coding for probes sensitive to peroxide (HyPer) and pH (both HyPer and SypHer). In this set of experiments, we used samples from 11 ALS patients (9 sALS and 2 fALS) with an average ALSFRSr 38.0 ± 1.6, aged 58.1 ± 3.6 years and IgG from age-matched control subjects (3 healthy and 2 disease controls, Table 1). Four ALS IgG (36.36%; three sALS and one fALS) induced acute rise in HyPer fluorescence intensity immediately after the application of IgG that continued to increase throughout the 5 min period, decreased slowly during the wash period, and rose again during the application of 100 µM H_2_O_2_ (Figures 6A–D, red traces). HyPer is a very sensitive indicator of peroxide, but since it is also sensitive to pH changes, control experiments were done with the same ALS samples on BV-2 cells transfected with SypHer that reacts only to the pH changes (peroxide sensing part in the plasmid is mutated). Interestingly, all four ALS samples induced an increase in SypHer intensity in the similar manner (although with the lower averaged amplitude), the intensity decreased during the washing period, and as expected, no change in fluorescence intensity could be detected in response to 100 µM peroxide (Figures 6A–D, blue traces). None of the control IgGs induced changes in HyPer intensity (example shown in Figure 6E). Since both plasmids have the same spectral characteristics, their response could be evaluated only on separate coverslips with cells, so we wanted first to evaluate the similarity of response of these two sensors to the same pH perturbation, induced by 30 mM NH_4_Cl. As expected, ammonium-chloride induced a very sharp increase in the fluorescence intensity (indicating increase in the cytosolic pH) followed by a slow decay during the stimulation with 30 mM NH_4_Cl (Figure 6F). The removal of ammonium-chloride was accompanied by sharp decay in fluorescence intensity (indicating decrease in cytosolic pH) that slowly returned to the baseline (Figure 6F). The amplitudes of maximal normalized fluorescence intensity in response to alkalization were measured in both cases of transfected cells, with HyPer (0.48 ± 0.02; *n* = 4) and SypHer (0.55 ± 0.03; *n* = 6), and the analysis showed no difference between the two groups (two-tailed *t*-test, *p* = 0.145; Figure 6G). Since the two sensors react in the same manner to pH changes, the attribution of peroxide to HyPer intensity changes could be determined by subtraction of SypHer from HyPer intensity. By comparing the intensities in the fifth minute after ALS IgG application (maximal measured intensity), the difference between HyPer and SypHer was greater than 10% only in the case of sample #7 (Figure 6B, 17% difference).

In addition, several experiments were conducted with carboxy-H2DCFDA, a general ROS indicator. In this set of data, we used three ALS samples and one control (see Table 1 for details). One ALS and one control IgG induced slow rise in the fluorescence intensity of the ROS probe (Figure 6H). Note that the increase in fluorescence intensity started later for the control IgG (Figure 6H, orange trace), and remained on the same level during the washing step, while the generation of ROS continued even during washing in the case of ALS IgG (Figure 6H, green trace). The amplitudes of ALS- and control IgG-induced normalized fluorescence intensities (reflecting ROS generation) are statistically different throughout the application and wash period (two-tailed *t*-test on values in the same time points). Linear regression analysis showed also the difference in slopes during the IgG application (*p* < 0.0001; *F* = 347.42, DFn = 1, DFd = 19598). Two of the ALS samples did not induce any changes (data not shown).

## Discussion

It is now widely accepted that the immune system has a role in ALS. Thus, a persistent and prominent activation of innate (e.g., complement activation) and adaptive (e.g., IgG secretion) immunity was noted, followed by T lymphocytes of the helper/inducer (CD4+), and in later disease phase, by cytotoxic/suppressor (CD8+) subtypes (4, 49, 50). A large body of experimental studies also indicates the specificity of ALS IgGs and their effect on neuronal and non-neuronal cells (21, 25, 51, 52). However, the binding site of ALS IgGs and the triggering mechanism remains elusive. As to the binding of IgG, there have been past attempts to ascribe it to Ca^2+^ channels (53–56) and the binding was evidenced in the presynaptic space of the motor plate (56). In addition, we and others have suggested a Ca^2+^ signaling mechanism underlying the ALS IgG effect (19, 21, 56, 57). Nevertheless, recently it has also been shown that ALS IgG can bind to the CD16 receptor on microglia or lymphocytes as well as to the immune synapse between the microglia and the neuron (58, 59).

Numerous studies demonstrate that microglial cells in the spinal cord and in the cerebral cortex become activated with the disease progression (2, 60, 61), which is now, along with astrocyte activation, considered to be one of the main characteristics of ALS (62). In this study we showed that ALS IgGs can induce a release of proinflammatory factors, ROS production and oxidative stress and upregulate antioxidative system in the microglial BV-2 cell line, which has been shown to be an appropriate experimental model for *in vitro* studies of cellular mechanisms of microglial activation (63, 64).

### BV-2 Microglia Releases TNF-α in Response to ALS IgG

TNF-α is a proinflammatory cytokine that is released in large amounts by microglia in pathological conditions, and this *de novo* production is considered to be an important feature of neuroinflammatory response associated with neurodegenerative diseases (65). While an earlier study has shown that intraperitoneal injection of ALS IgG in mice resulted in the activation of microglia (66), a more recent study detected an increased release of TNF-α in the spinal cords of animals inoculated with ALS IgG (24). Our experiments are in line with previous finding, as we demonstrated a significant release of TNF-α after 24 h treatment of BV-2 cells with ALS IgG (Figure 4A), and stress out the contribution of microglia to the ALS IgG-induced inflammatory response. Elevated levels of TNF-α could potentiate glutamate excitotoxicity, a well-established fact in ALS, either influencing the balance of excitatory and inhibitory receptors on neurons, or indirectly by inhibiting glutamate uptake by astrocytes (65). Our immunocytochemistry experiments showed that both ALS IgG and control IgG have similar binding patterns on the cell surface of the BV-2 cells (Figure 5). Therefore, we excluded the possibility that effects on TNF-α release, as well as the other results presented in this study, were due to direct intracellular effects of these humoral factors. However, further experiments have to be carried out in order to reveal the differences in the signaling pathways of ALS IgG and control IgG. In our study, no difference was detected in gene expression level of TNF-α (Figure 4B), which is probably due to a relatively short treatment with IgG (4 h). Our previous study on BV-2 cells has shown that LPS induced changes in TNF-α gene expression (67), which is in disagreement with this finding. However, this difference may be due to different types of activator molecules and therefore possible differences in signaling pathways. Nevertheless, we were able to detect a dramatic increase in TNF-α gene expression of BV-2 cells even after 4 h of treatment with IgG from a fALS patient (sample #3, Table 1) with mutation in SOD1 and ALSFRSr 45 (red dot, Figure 4B). This outlier result may indicate a potential functional difference in IgGs from sALS and fALS patients that prompts a future study toward a differential disease marker. Notably, it was found that a variant of exogenous mutant SOD1 (G93A) protein could also induce a release of TNF-α in microglia that could induce toxicity to motor neurons *via* TLR and CD14 pathways (68). Taken together, further investigations are necessary in order to determine the signal cascade(s) that ALS IgG initiates in microglial cells, and potential differences or similarities in the mechanism(s) of action of sALS and fALS IgG.

### ALS IgG Have Potency to Induce Oxidative Stress in BV-2 Cells

As a further proof of microglial humoral activation the results of this study show that the NO production was significantly increased in BV-2 cells by ALS IgG (Figure 1B). These results are in agreement with a previous study on the ALS animal model showing that the disease progression is followed by an increase in the number of microglial cells expressing inducible nitrate oxide synthase (69). While the effect of NO might be overestimated *in vitro* where there is no drain for this molecule, NO diffuses to red blood cells in tissue within seconds, where interacts with oxyhemoglobine (70). Nevertheless, while NO is moderately toxic, high concentrations may out-compensate SOD, and it may react with the superoxide anion to form peroxynitrite, that can selectively oxidize certain biological macromolecules, a mechanism pointing to a common cause of *in vitro* motor neuron death (71). In addition, we observed a significant increase of the index of lipid peroxidation (MDA production; Figure 1B), another confirmation of our hypothesis that ALS IgG can induce oxidative stress, that was in agreement with results obtained *in vivo* and *ex vivo* from brainstem and hippocampus of hSOD1(G93A) rats (32). Wu et al. (61) showed that NOX is activated in spinal cords of ALS patients and animal model of the disease, where NOX driven oxidant products from activated microglial cells impair the survival pathways of motor neurons, linking oxidative stress with neuronal death in ALS. Interestingly, our study has found increased NOX2 expression even after 4 h of treatment with one fALS (SOD1D90A) IgG (red dot Figure 3D), stressing out the need for additional experiments that might define the mechanism(s) underlying potential functional differences between familial and sporadic form of the disease.

### Antioxidative System Is Upregulated after Treatment with ALS IgG

This study shows that the activity of MnSOD and Cu/ZnSOD was elevated after the treatment with ALS IgG (Figures 2A,B), as well as CAT (Figure 2C), but without a change in gene expression after 4 h treatment (Figure 3B). As a consequence of oxidative stress in an *in vitro* model of microglial activation with LPS, protein expression of MnSOD and Cu/ZnSOD was also increased (72). Therefore, we can assume that the antioxidative defense in BV-2 cells is upregulated as a result of the microglial activation and/or oxidative stress induced by ALS IgG. Analysis of gene expression of MnSOD after 4 h treatment did not show any change, although again the same outlier sample from the fALS patient (score 45) induced an increase in gene expression. Interestingly, as this fALS patient has mutated SOD1, these data are in agreement with elevated activity of MnSOD in the brainstem and hippocampus of hSOD1G93A rats, already in the presymptomatic stage (32). This could be explained as a compensatory mechanism of antioxidative defense when SOD1 activity is reduced due to mutation, and oxidative stress is most probably increased. In addition, we found that the total glutathione level was significantly decreased due to ALS IgG treatment compared with untreated cells (Figure 2F). A characteristic of *in vitro* microglial stimulation with LPS and IFNγ, as well as in oxidative stress is a decrease in total glutathione level (73, 74). These data underline that ALS IgG induces oxidative stress in BV-2 cells. Regarding the antioxidative defense an upregulation was observed as a rise in GR activity following the ALS IgG treatment (Figure 2E). On the other hand, we observed a significantly decreased GPx activity (Figure 2D) compared with untreated cells, and there was no change in the gene expression level compared with both groups (Figure 3C). One might speculate that while the increase in the GR activity reflects upregulation of antioxidative system, the decrease in GPx activity might be the consequence of the decrease in substrate, as the total glutathione levels decrease. The role of GPx in ALS, however, is controversial because different studies showed that its activation and expression in spinal cord and cerebral cortex of patients can be decreased (75), without change compared with the control group (76) or not detectable at all (77).

We were unable to detect a change in gene expression for NOX2 after the treatment with ALS IgG (Figure 3D), which is in disagreement with results that show an upregulation of NOX2 in an animal model with the disease progression (61). The possible explanation of this discrepancy could be that it might take more than 4 h of ALS IgG treatment for the change in NOX2 expression or any other gene considered in this study. It is also worth mentioning that the sample from the fALS patient (with mutation in SOD1D90A, and ALSFRSr of 45) induced a rise in gene expression of NOX2, as well as of MnSOD and TNF-α, again indicating a possible differential marker for sALS and fALS that needs to be further studied.

### Acute ROS Production Is Induced by ALS IgG

Our imaging experiments on single cell responses to acute application of ALS IgGs revealed an expected heterogeneity of reactions, having in mind the heterogeneity of sALS patients in terms of, e.g., clinical course, disease duration and response to pharmacological treatment. The common denominator of all experiments is that roughly one third of ALS samples show a well-defined rise in the signal for pH-sensitive (SypHer, HyPer) and peroxide sensitive (HyPer) genetically encoded indicators, as well as for ROS-sensitive probe (Carboxy-H2DCFDA). Although not significantly different from pH change in all studied cases, the peroxide response with HyPer was always on top of pH-specific SypHer signal. The ALS IgG-induced ROS generation was significantly higher than control IgGs-induced, as confirmed with carboxy-H2DCFDA. The difference in ROS generation during the washout period between those two types of IgG (Figure 6G, indicated by arrows) might indicate stronger or more specific binding of ALS IgG to the still unknown BV-2 membrane antigen. Nevertheless, alkalization of the cytoplasm induced by ALS IgGs is also a sign of antioxidative defense by way of proton extrusion (78) where NHE1 was suspected as a key transporter (48). We have thus, checked the gene expression for NHE1 after 4 h ALS IgG-treatment, but were unable to detect a change in mRNA (Figure 3E), possibly due to the short treating period. Hence, additional experiments would be needed to confirm or exclude the role of NHE1 in the activation of microglia by ALS IgG.

The present study in an *in vitro* setup provides a missing link between the oxidative stress in ALS and many different actions of humoral factors in the underlining neuroinflammation. It focuses on microglial cells as key players of the inflammatory response and highlights ALS IgG-induced ROS generation as the trigger of activation of initially healthy microglia. Revealing the ALS IgG signaling cascade in microglial cells could offer valuable molecular biomarkers and/or potential therapeutic targets. Namely, such biomarkers could be followed in microglial cells from patients (i.e., derived form inducible pluripotent stem cells) under no or standard stimulation (e.g., with ATP or H_2_O_2_) or as shown here, healthy cells (preferably of human origin) could be used to test ALS IgGs for better disease profiling.

## Ethics Statement

Sera from patients at the Neurology Clinic, Clinical Center of Serbia were collected for routine clinical examination with informed patient’s consent in accordance with The Code of Ethics of the World Medical Association (Declaration of Helsinki) for experiments involving humans. The protocol was approved by the Ethics committee of the Clinical Center of Serbia (no. 1985/5).

## Author Contributions

All authors contributed sufficiently for being listed as authors of this article. Study design: PA and MM. Patient selection and diagnostics: ZS. IgG preparation: IŽ. Cell culture preparation and treatments: MM, KM, and MD. Performed PCR and ELISA assays: KM, IB, and IL. Biochemical assays: IS. Immunocytochemistry: KM, DB, and MM. ROS imaging: MM, KM, MD, and RG. Data analysis and interpretation: MM, PA, KM, IB, DB, and IL. Writing of the article: MM, KM, and PA.

## Conflict of Interest Statement

The authors declare that the research was conducted in the absence of any commercial or financial relationships that could be construed as a potential conflict of interest.
